# Investigation of the Nonlinear Optical Properties of Silk Fibroin (SF) Using the Z-Scan Method

**DOI:** 10.3390/ma18051052

**Published:** 2025-02-27

**Authors:** Georgi Yankov, Victoria Atanassova, Stefan Karatodorov, Radostin Stefanov, Krum Shumanov, Ekaterina Iordanova, Albena Daskalova, Liliya Angelova, Emil Filipov

**Affiliations:** 1 Institute of Solid State Physics, Bulgarian Academy of Sciences, 72 Tsarigradsko Chausse, Blvd., 1784 Sofia, Bulgaria; 2 Institute of Electronics, Bulgarian Academy of Sciences, 72 Tzarigradsko Chaussee, Blvd., 1784 Sofia, Bulgaria; albdaskalova@gmail.com (A.D.);

**Keywords:** silk fibroin, *z*-scan method, femtosecond laser pulses, nonlinear optical refractive index, multiphoton absorption coefficient, optical transparency

## Abstract

Silk fibroin (SF), the primary protein in silkworm silk, has emerged as a promising organic nonlinear optical material due to its unique combination of optical transparency, biocompatibility, and environmental sustainability. In this study, we investigate the nonlinear optical properties of SF thin films using the *z*-scan technique with femtosecond laser pulses (35 fs, 800 nm, 1 kHz). Our results reveal a strong self-defocusing effect (negative nonlinear refractive index) and significant multiphoton absorption, demonstrating SF’s tunable nonlinear response. Additionally, optical transmittance measurements confirm SF’s partial transparency in the deep UV region, enhancing its potential for second-harmonic generation (SHG) and efficient light frequency conversion. These findings address a key knowledge gap in nonlinear optics, positioning SF as a versatile biopolymer for advanced photonic applications.

## 1. Introduction

Nonlinear optics lay the foundations of cutting-edge photonics technologies, enabling remarkable scientific advancements by allowing photons to complement or even replace electrons in many applications traditionally dominated by microelectronics [1]. Nonlinear optical processes such as frequency conversion, optical switching, and modulation play an essential role in photonics [2]. Over the past few decades, these processes have attracted significant attention across diverse fields, including telecommunications, laser technologies, optoelectronic devices, etc. [3,4,5,6,7,8,9].

In recent years, organic and polymer-based systems have emerged as a promising class of nonlinear optical materials [10,11,12,13,14]. Their development, particularly for achieving efficient second and third-order nonlinearities, has been a focal point of research [15]. These materials offer large optical nonlinearities alongside excellent mechanical, chemical, and thermal stability, positioning them as strong candidates for optoelectronic device manufacturing [1]. Some organic materials even demonstrate nonlinear coefficients much larger than those of conventional inorganic dielectrics and semiconductors [16,17,18]. Another reported advantage is their ultrafast response times, often on the femtosecond timescale, which favors fast optical switching and sensing applications [15,19].

Meeting the high demands of modern optoelectronic technologies requires a deep understanding of the physical mechanisms governing nonlinear optical responses. Organic polymers generally exhibit much larger optical nonlinearities compared to traditional inorganic materials due to their unique molecular structures, electronic properties, and flexibility in molecular engineering. The nonlinear optical response is largely attributed to the presence of conjugated π-electron systems, which enable delocalized electrons to respond strongly to electric fields and exhibit high polarizability. This delocalization results in significant second-order (χ^2^) and third-order (χ^3^) nonlinear optical susceptibilities, making organic polymers highly effective for nonlinear optical applications [1,20].

Accurate characterization of the emerging materials’ microscopic and macroscopic nonlinear optical properties is also inevitable. Organic and polymeric systems, in particular, allow tuning and optimization of nonlinear optical properties through molecular design while maintaining thermal, mechanical, and chemical stability. This adaptability offers significant advantages in terms of processability compared to traditional dielectric crystals and semiconductors [1,21].

**Figure 1:**
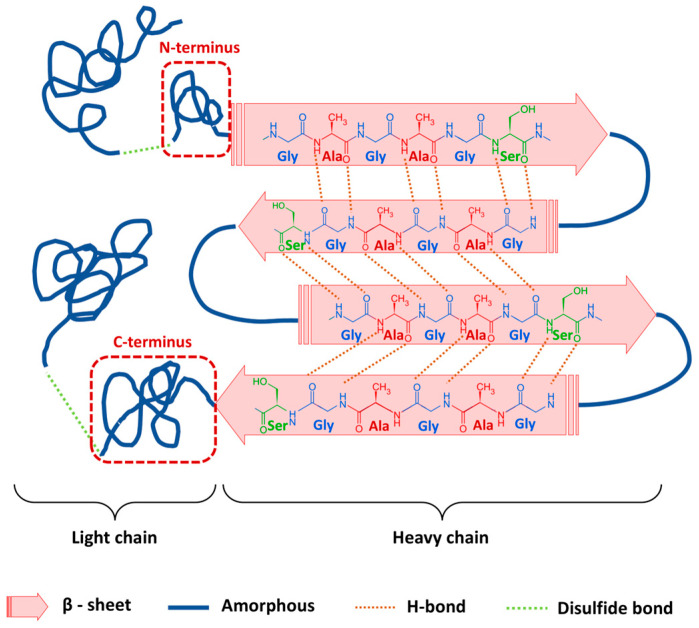
Chemical structure of silk fibroin (SF). The hydrophobic heavy (H-) chain forms ordered β-sheet nanocrystals stabilized by hydrogen bonds, while the hydrophilic light (L-) chain consists of random coils and α-helices with variable hydrogen bonding. The two chains are linked by disulfide bonds.

An example of such a biocompatible polymer molecule is Silk fibroin (SF), the primary protein in Bombyx mori silkworm silk, comprising over 70% of its weight. SF is a high-molecular-weight biopolymer with a semi-crystalline structure. The characteristics of SF emerged from the combination of peptides and polypeptides. Silk fibroin is a protein-based material, with minor constituents such as lipids and polysaccharides. Its structure comprises sequential crystalline hydrophobic heavy (H-) chains that form ordered antiparallel β-sheet nanocrystals stabilized by strong hydrogen bonds, as well as amorphous hydrophilic light (L-) chains composed of random coils and α-helices with variable hydrogen bonding. Additionally, it includes a small glycoprotein known as P25. The H-chain and L-chain are linked via disulfide bonds. The amino acid sequences of the H-chains predominantly feature glycine (Gly) in various repetitive patterns (Figure 1) [22,23,24]. This unique structure gives SF exceptional mechanical properties, including high toughness, tensile strength, and surface smoothness, surpassing the properties of many other biopolymers. It is also biocompatible, biodegradable, and resorbable, and offers versatile processability, functional adaptability, and thermal durability [23,25,26,27,28,29,30].

SF can be extracted from Bombyx mori silkworm silk through a water-based method and can be efficiently prepared as an aqueous solution that facilitates the fabrication of versatile structures such as hydrogels, fibers, ultra-thin and thick films, 3D porous structures, controlled-release coatings, etc. [31,32,33,34,35]. Fibroin-hybrid systems based on modifications of the chemical structure of the protein with polymers and inorganic compounds enhancing the SF intrinsic properties have been studied recently [36]. These characteristics are extremely valuable in biomedical applications where silk fibroin is a highly recognizable material, such as tissue engineering, health monitoring sensors, diagnostics, wound healing, drug delivery, etc. [30,34,37,38,39]. There are some studies on the use of electrospun silk for personal protection, such as air and water filtration and thermal and humidity regulation [40]. It has been explored in the field of energy harvesting, different kinds of flexible sensors, and other flexible electronic devices [32], as well as in the field of food industry [41,42]. Moreover, as a natural and environmentally sustainable biopolymer, SF aligns with the emerging concept of green devices, further enhancing its relevance in modern applications [43].

Silk fibroin has emerged as a promising material for optical and photonic applications due to its broad transparency range from ultraviolet to near-infrared, low scattering coefficient, hierarchical structure, tunability, and ease of processing. Furthermore, the structural characteristics of silk fibroin can be adapted to adjust its index of refraction, thus enabling optimized designs for optical elements such as sensors and waveguides [44,45]. Recent advances have showcased its application in structural color materials, optical fibers, diffractive optical elements, bio-lasers, plasmonic devices, and metamaterials. The capacity of silk fibroin to undergo controllable conformational transitions allows for the creation of reconfigurable, tunable, and transient optical systems. Additionally, its biocompatibility and biodegradability make it a sustainable alternative to traditional synthetic optical materials, thereby opening new possibilities for implantable and bioresorbable photonic devices [26,46,47,48,49,50,51,52,53].

Although silk fibroin (SF) is a well-known material for its biomedical and optical applications, it has also attracted interest in nonlinear optics. However, previous studies on its nonlinear optical response are scarce. Applegate et al. [54] investigated the three-photon absorption properties of SF and Lee et al. [55] further explored the third-order nonlinear susceptibility of SF demonstrating enhanced nonlinear optical properties through a modification of SF’s molecular structure, particularly by increasing the β-sheet content. SF also shows significant potential as a medium for light frequency conversion, as Zhao et al. [56] demonstrated second harmonic generation (SHG) signals in spider silk. Many conventional materials used in nonlinear optics are well-characterized in terms of their third-order nonlinear susceptibility (χ^3^), nonlinear refractive index (n_2_), and nonlinear absorption coefficient (β). In contrast, SF’s nonlinear refractive index and multiphoton absorption coefficients have not been extensively measured under femtosecond irradiation, leading to a serious knowledge gap in nonlinear optics.

This study investigates the nonlinear optical properties of silk fibroin (SF) thin films using the *z*-scan method with femtosecond laser pulses (35 fs, 800 nm, 1 kHz). By quantifying the nonlinear refractive index (n_2_) and multiphoton absorption coefficient (β), we provide the first detailed analysis of SF’s nonlinear response under intense laser fields at these conditions. Our findings address a major knowledge gap in nonlinear optics, demonstrating SF’s strong self-defocusing behavior and high multiphoton absorption. Furthermore, we investigate SF’s potential for second-harmonic generation (SHG) due to the partial optical transparency in the deep UV region observed in this study. These characteristics position SF as a promising biopolymer for advanced photonic applications.

## 2. Materials and Methods

### 2.1. 2D Silk Fibroin Thin Film Samples

**Figure 2:**
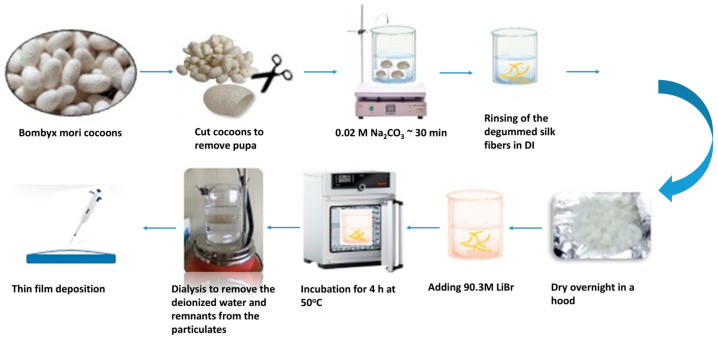
Schematic representation of the basic steps for silk fibroin extraction.

Thin solidified silk fibroin (SF) films were prepared to conduct the investigation. To extract and purify fibroin from the silk filaments of the silkworm *Bombyx mori* cocoons, already established protocols for silk fibroin extraction and purification were used [57], further modified after a series of experiments to obtain an optimal “medium” for bio application. Briefly, the optimized fibroin extraction procedure involves the following steps: silk fibroin is extracted and purified from *Bombyx mori* cocoons (Institute of Bombyx, Vratsa, Bulgaria). Production of silk fibroin (approximately 7–9% in dH_2_O) consists of degumming with sodium carbonate and lithium bromide (Sigma-Aldrich^®^, Munich, Germany). The procedure involves three main steps: first, the preparation of silk cocoons by removing the molecule from the cocoon and peeling off the inner layer. Second, degumming is done by boiling the cocoon material in 0.02 M Na_2_CO_3_ (Valerus, Sofia, Bulgaria), washing and drying the resulting degummed silk. This step is crucial for removing the protein sericin, which protects the fibroin in silk fibers. Finally, dissolve the sericin-free silk in a 9.3 M LiBr solution for 3 h at 60 °C. The dissolved silk was then dialyzed against water for 48 h (water changed at 2, 4, 6, 14, 24, and 48 h) and centrifuged for 10 min at 4618× *g* (Figure 2). The resulting SF (7.26% *w*/*v*) solution was used to prepare 2D thin films (2 × 2 cm, 110 µm thick) by applying 700 µL of the resulting solution to glass slides. The overall thin film thickness, achieved after drying, was 110 μm. The measurement was performed via a coating thickness gauge VA 8042 coating meter (Shenzhen, China) and was averaged over 10 separate measurements.

### 2.2. Preliminary Characterization

Before performing the *z*-scan analysis, full morphological and chemical characterization of the created silk-based thin films was conducted. For this purpose, Scanning Electron Microscopy (SEM) and Energy-Dispersive X-ray (EDX) Spectroscopy (SEM—TESCAN/LYRA/XMU, Fuveau, France), 3D profilometry (3D Optical profiler Zeta-20 KLA, Milpitas, CA, USA), and Atomic Force Microscopy (AFM) (MultiMode V, Veeco Instruments Inc., New York, NY, USA), as well as Fourier Transform Infrared (FTIR) spectroscopy (IR Affinity-1, Shimadzu, Kyoto, Japan), were performed. The linear optical transmittance of the samples over the UV-Vis-NIR spectral region (200–2200 nm) was measured via spectrophotometer Perkin Elmer Lambda 1050 (PerkinElmer, Shelton, CT, USA). All analyses performed were conducted concerning the approved European standards, following the general recommendations in ISO 16700:2016 Surface chemical analysis [58] and ISO/TS 80004-6:2021 Nano-object characterization [59].

### 2.3. Z-Scan Method

The *z*-scan method is a standard technique for the fast and simple determination of the nonlinear refractive index *n*_2_ and the nonlinear absorption *β* in solids, liquids, and liquid solutions with high accuracy [60]. It was proposed for the first time by Sheik-Bahae et al. [61]. The main idea of the measurement consists of moving the sample longitudinally around the focal plane of a Gaussian laser beam and then, owing to optical nonlinear response (self-focusing or defocusing and multi-photon absorption), the incident intensity distribution promotes changes in the measured nonlinear optical coefficients of the material, which alters the beam propagation. The *z*-scan signal is a function of the transmitted light intensity distribution in the far field, which varies with the sample position around the focal plane [62]. It is usually displayed as a function of the ratio *z*/*z*_0_ of the transmittance, where z is the distance of the sample from the focal plane in [m], *z*_0_ = (*πω*_0_^2^)/*λ* is the diffraction length of the beam in [m], *ω*_0_ is the beam radius at the waist in [m], and *λ* is the laser wavelength in [m]. The transmittance of the medium is measured with closed or open aperture, which gives the information about *n*_2_ in [m^2^/W] or *β* in [m/W], respectively.

The nonlinear refractive index can be calculated through the following Formula (1):

(1) $$n_{2}=\frac{\lambda ∆T_{p-v}}{4L}\frac{\pi \omega_{0}^{2}}{2P},$$

where *L* is the optical length of the measured sample in [m], Δ*T_p−v_* is the ratio change of the transmittance function ‘peak to valley’ (dimensionless), and *P* is the laser peak power in [W].

The nonlinear absorption coefficient can be calculated through Formula (2):

(2) $$\beta =\frac{2\pi ∆T\omega_{0}^{2}}{2PL}\;.$$

The detailed mathematical approach is described elsewhere [63]. During the calculations, the aforementioned SI units for all variables in Equations (1) and (2) are considered.

### 2.4. Z-Scan Experimental Setup

**Figure 3:**
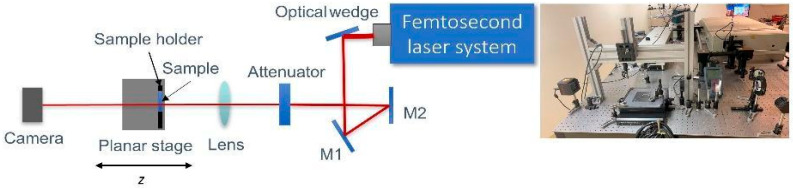
A scheme of the experimental setup for *z*-scan measurements (**left**) and an image of the actual setup (**right**).

The experimental setup for the *z*-scan method was based on the original concept published in [61], but it was modified by incorporating a laser beam profiler camera instead of a standard aperture, and a femtosecond laser was used. A schematic representation of the experimental setup is shown in Figure 3. The experiments were conducted using a titanium-sapphire femtosecond laser system Spitfire Ace (Spectra-Physics, Milpitas, CA, USA) with a wavelength of *λ* = 800 nm, a repetition rate of *υ* = 1 kHz, and a pulse duration of *τ* = 35 fs. This setup provides exceptional precision and resolution, making it ideal for investigating nonlinear optical phenomena. Additionally, the application of ultra-short pulses minimizes cumulative heating effects caused by closely spaced in-time consecutive pulses, which could otherwise influence the measurement of *n*_2_. The laser beam is guided through an optical system composed of an optical wedge attenuating the laser energy, a pair of reflective mirrors (M1 and M2), an additional set of energy attenuators, and a lens with a focal length of *f* = 250 mm. The incident energy measured before the lens was *E* = 412 nJ, which was experimentally found to be the optimal laser energy for performing the *z*-scan measurements.

The samples were mounted on a holder attached to a Planar DL two-axis mechanical bearing direct-drive linear stage system (Aerotech, Pittsburgh, PA, USA), which was utilized for precise positioning of the SF samples. This system, with travel ranges of 100 mm × 100 mm, an accuracy of ±0.4 µm, a flatness of ±1 µm, and a maximum speed of 500 mm/s, allows for fast and precise movement of the samples along the *z*-axis within the focal plane of the lens. The measurement step size used was 0.3 mm.

The laser beam profile was detected using a laser beam profiler camera, model BGP-USB-L11059 (Ophir Optronics, Jerusalem, Israel). The camera captures and analyzes wavelengths ranging from 190 nm to 1100 nm. It is a silicon CCD camera and features an image format of 35 mm × 24 mm, high-speed electronic shutter, dynamic range of 59 dB, true dynamic resolution, beam sizes in the range 90 μm–23.8 mm, sensor elements of 4008 × 2672, frame rate (full resolution) of 3.1 fps, camera sensitivity 9.8 dB, good signal-to-noise ratio. This camera is designed for precise laser beam analysis, offering high resolution and speed. The camera was positioned at a 720 mm distance from the lens and 470 mm from its focal point.

The measurements were conducted according to widely accepted experimental procedures in scientific literature.

## 3. Results

**Figure 4:**
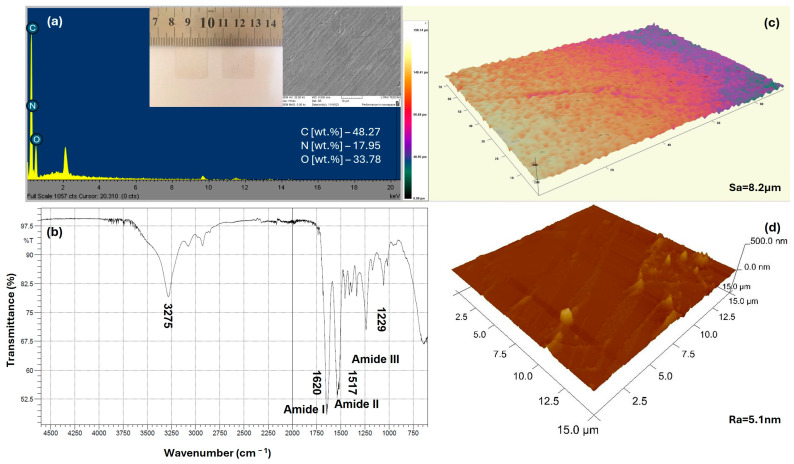
Multicomponent image, showing results of the performed analysis on the silk samples: (**a**) EDX spectrum [wt.%], optical and SEM image (at 5.00 kx magnification); (**b**) FTIR transmittance (%) spectrum in the range of 750–4500 cm^−1^; (**c**) 3D surface profile (taken at 20×) and (**d**) AFM 3D image at 15 × 15 μm with the corresponding Sa** and Ra* values in micro- and nano-range. * Ra—mean value of the variation of the surface height value from the median baseline. ** Sa—the extension of Ra to a surface area.

The preliminary characterization of the morphological properties and the chemical composition of the created silk-based thin films are presented on the multicomponent Figure 4 below:

As can be seen from the results presented, apart from being very thin (around 110 μm) the silk-based samples are characterized by homogeneous morphology (SEM) and surface area (Sa) and line (Ra) roughness in micro- (3D-profilomery) and nano-diapason (AFM), respectively. Apart from C, O, and N, no uncommon elements for the silk polypeptide are detected (EDX) [64]. All characteristic transmittance peaks arising from the peptide bond –CONH– of the amide groups of the protein are positioned at the right places as follows (FTIR): amide I: C=O stretching at 1620 cm^−1^, amide II and amide III: N–H bending, and in-phase combination of C=O bending and C–N stretching at 1517 cm^−1^ and 1229 cm^−1^, respectively [65].

A crucial component of the experimental procedure involved measuring the nonlinear optical properties of various samples. Initial calibration measurements were performed on fused quartz samples with known thicknesses (0.5 mm, 1 mm, and 2 mm) to ensure the accuracy of the measurement system. These calibration results established a reliable baseline for calculating average values and assessing the repeatability of the data. The findings indicated no significant variation across the different thicknesses.

**Figure 5:**
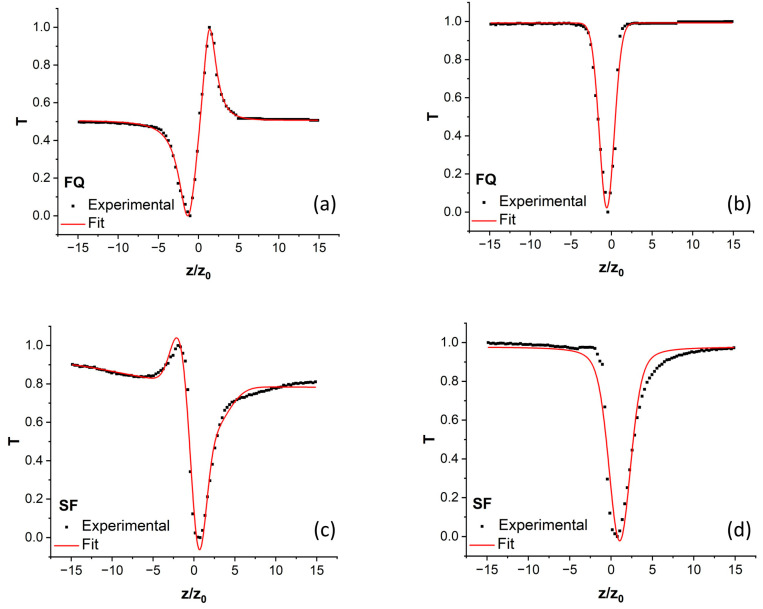
Z-scan measurements of: (**a**) nonlinear refractive index of fused quartz (FQ); (**b**) nonlinear absorption coefficient of fused quartz; (**c**) nonlinear refractive index of silk fibroin (SF); (**d**) nonlinear absorption coefficient of silk fibroin.

Figure 5 illustrates the *z*-scan measurement curves (dots) for the nonlinear optical properties of fused quartz (Figure 5a,b) and silk fibroin (Figure 5c,d). The corresponding theoretical fits (red lines), derived from the equations proposed by Sheik-Bahae et al. [61], are also displayed.

For fused quartz, the nonlinear refraction curve features a pre-focal transmittance minimum (valley) followed by a post-focal transmittance maximum (peak), symmetrically positioned around the focal point (*z* = 0). This pattern confirms that the nonlinear refractive index (*n*_2_) of fused quartz is positive, consistent with previously published reports [66]. Consequently, fused quartz exhibits a self-focusing effect.

In contrast, the nonlinear refraction curve for silk fibroin shows a transmittance peak before the focal point and a valley afterward, resulting in an asymmetric pattern. This indicates that the nonlinear refractive index (*n*_2_) of silk fibroin is negative, signifying a self-defocusing effect. The asymmetry is attributed to stronger nonlinear absorption, which diminishes the peak and amplifies the valley of the transmittance [61].

The contrast in the optical nonlinearities of the two materials is highlighted by the calculated nonlinear optical coefficients, as shown in Table 1. The values obtained for fused quartz align with those reported in [67] (p. 10). The author notes that the *z*-scan measurement of fused silica, obtained with a femtosecond laser operating at 800 nm with a 37-fs pulse duration (conditions closest to those of our experimental setup), yielded a value of 5.62 × 10⁻¹⁶ cm^2^/W. This result is in good agreement with the value obtained in our experiment. In comparison, the SF thin films demonstrated significantly enhanced nonlinear optical properties. Specifically, the nonlinear refractive index of silk fibroin was found to be an order of magnitude higher than that of fused quartz, while the multiphoton absorption coefficient was two orders of magnitude greater.

**Figure 6:**
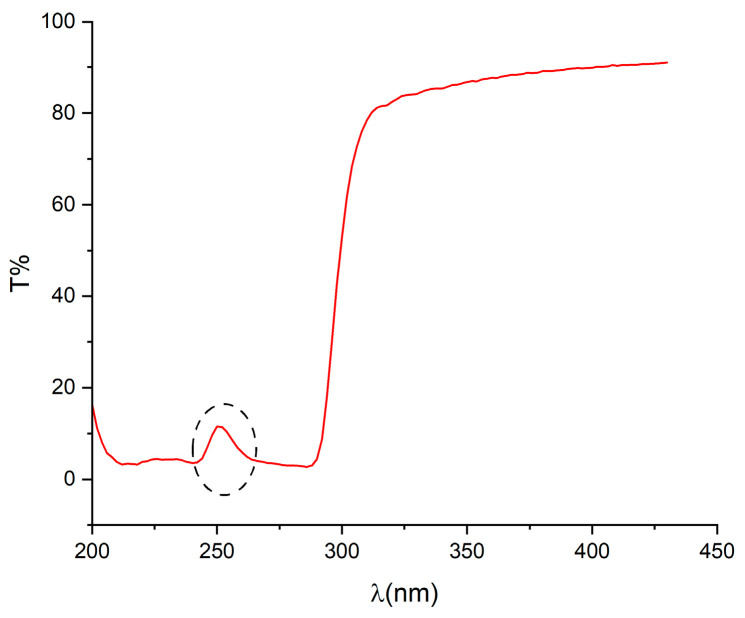
Linear optical transmission (T) of silk fibroin in the wavelength range of 200–450 nm. The characteristic transmittance peak around 250 nm is circled with a dashed line.

The linear optical transmittance of SF shows another potential for its nonlinear optical applications. Studies have shown that SF exhibits high optical transparency, exceeding 90% transmittance, across the 300–2000 nm spectral range [44]. This characteristic feature makes SF suitable for use as an optical substrate in various photonics and optical applications [46,48]. Figure 6 illustrates the optical transmittance of SF in the 200–450 nm spectral range. The observed transmittance curve aligns well with reports in the literature [44,68]. The decrease in optical transmittance in the 200–300 nm region is attributed to UV absorption by the aromatic amino acids present in the SF structure. The transmittance peak around 250 nm (circled with a dashed line in Figure 6) is associated with interactions involving these aromatic residues [68].

## 4. Discussion

The observed nonlinear optical response of SF is attributed to its specific chemical structure. It comprises a sequence of amino acids, including, for example, aromatic residues in the structure of tyrosine (Tyr) and tryptophan (Trp). Although these aromatic amino acids are present in relatively small concentrations (5.3% and 0.5%, respectively), they are well-known for their optical responsiveness [55]. In the research conducted by Applegate et al. [54] tryptophan has been identified as a key contributor to the nonlinear response of SF. Furthermore, the large optical nonlinearity of SF can be plausibly linked to the delocalized π-electron systems in these aromatic residues and the complex intermolecular interactions within the biopolymer matrix.

The negative nonlinearity (self-defocusing) and significant multiphoton absorption observed in our study contrast with the findings of Lee et al. [55]. Their measurements on a SF film with 200 nm thickness revealed a positive nonlinear refractive index (self-focusing) and negligible multiphoton absorption, while our results on a film with a 110 μm thickness demonstrate self-defocusing and strong multiphoton absorption. This discrepancy highlights the strong influence of material thickness on the nonlinear optical response. Thicker films provide larger interaction volumes, enhancing multiphoton absorption [69]. Higher nonlinear absorption contributes to self-defocusing effects and asymmetry in the nonlinear refraction curves observed in our study. These findings suggest that SF’s nonlinear response can be tuned through fabrication parameters, allowing precise control over its optical behavior and enhancing its versatility for a wide range of applications. The observed negative nonlinearity is particularly valuable for applications requiring high-intensity laser beam control, such as optical limiting [70], beam shaping [71], mitigation of filamentation in high-power lasers [72,73], formation of dark spatial solitons for optical signal processing [74], etc.

Previous studies have demonstrated that SF is an SHG-active material [56,75]. The optical transmittance that we measured in the 200–450 nm spectral range reveals a distinct transmission profile, characterized by partial transparency in the deep UV region. This unique optical behavior enhances SF’s potential for efficient light frequency conversion, particularly for SHG applications in systems operating with fundamental wavelengths in the visible spectrum by ensuring minimal absorption at both the fundamental and second-harmonic wavelengths. Furthermore, the ability to tailor SF’s optical properties through fabrication methods suggests its potential for broader frequency conversion applications, including third-harmonic generation and other nonlinear optical processes.

## 5. Conclusions

This study investigated the nonlinear optical properties of silk fibroin (SF) thin films by measuring the nonlinear refractive index (*n*_2_) and multiphoton absorption coefficient (*β*) using the *z*-scan technique with a femtosecond laser system. The results revealed a self-defocusing effect and strong multiphoton absorption in SF films, contrasting with previous findings that reported self-focusing and negligible multiphoton absorption. This difference emphasizes the critical role of material thickness in determining the nonlinear optical response, as thicker films enhance multiphoton absorption and, consequently, self-defocusing effects. These results indicate the potential for tailoring SF’s nonlinear properties through fabrication, enabling precise control over its optical behavior for diverse applications.

The high nonlinearities observed in SF are attributed to its molecular structure, specifically the presence of aromatic amino acids such as tryptophan. This is likely related to the delocalized π-electron systems in the residues of these aromatic amino acids and complex intermolecular interactions within the biopolymer matrix.

While there is enhanced absorption in the 200 to 350 nm spectral range, the notable transmittance peak at approximately 250 nm is advantageous for light frequency conversion applications, such as second harmonic generation (SHG) in systems that utilize a fundamental wavelength within the visible spectrum.

The unique combination of high nonlinearities, optical transparency, biocompatibility, and environmental sustainability makes SF suitable for diverse applications. Potential uses include optical limiting for laser safety, beam shaping, frequency conversion in photonic devices, and optical signal processing. The ability to tailor SF’s nonlinear optical properties through structural modifications further expands its potential for next-generation optoelectronic and photonic technologies.

## Figures and Tables

**Table 1:** Calculated values of the nonlinear optical coefficients for fused quartz and silk fibroin.

Material	*n*_2_ [m^2^/W]	*β* [m/W]
Fused quartz	5.57 × 10^−20^	6.4 × 10^−14^
Silk fibroin	8.8 × 10^−19^	9.1 × 10^−12^

## Data Availability

The original contributions presented in the study are included in the article, further inquiries can be directed to the corresponding author.

## Abbreviations

The following abbreviations are used in this manuscript:

SF	Silk fibroin
FQ	Fused quartz
UV-Vis-NIR	Ultraviolet-Visible-Near Infrared
SHG	Second harmonic generation
SEM	Scanning electron microscopy
EDX	Energy-Dispersive X-ray
AFM	Atomic Force Microscopy
FTIR	Fourier Transform Infrared
CCD	Charge-Coupled Device
Tyr	Tyrosine
Trp	Tryptophan
